# Microbial investigation of cleanability of different plastic and metal surfaces used by the food industry

**DOI:** 10.1007/s13197-023-05778-0

**Published:** 2023-07-06

**Authors:** Claudia Waldhans, Martin Hebel, Ulrike Herbert, Paul Spoelstra, Shai Barbut, Judith Kreyenschmidt

**Affiliations:** 1 Institute of Animal Science, University of Bonn, Katzenburgweg 7-9, 53115 Bonn, Germany; 2 Dr. Berns Laboratorium GmbH & Co. KG, Bendschenweg 36, 47506 Neukirchen-Vluyn, Germany; 3 Marel Poultry B.V, Handelstraat 3, 5831 AV Boxmeer, The Netherlands; 4 Food Science Department, University of Guelph, 50 Stone Road East, Guelph, ON N1G 2W1 Canada; 5 Department of Fresh Produce Logistics, Hoschschule Geisenheim, Von-Lade-Strasse 1, 65366 Geisenheim, Germany

**Keywords:** Cleanability, Conveyor belts, Food industry, Hygienic design, Nano materials, Stainless steel finishes

## Abstract

Different conveyor belt materials used by the meat and other food industries were compared, regarding their cleanability as bacterial reduction rates in relation to their surface topography. Eleven thermoplastic polymers, four stainless steels, and five aluminized nanostructured surfaces were investigated under laboratory conditions. Cleanings were conducted with water only, and with an alkaline foam detergent. Overall, scanning electron microscopy revealed remarkable differences in the surface topography of the tested surfaces. Water cleaning results showed that nanostructured aluminized surfaces achieved significantly higher cleanability rates compared to the eight thermoplastic surfaces, as well as the glass-bead blasted rough stainless steel. Thermoplastic surfaces showed overall low cleanability rates when cleaned with alkaline detergent, while stainless steel and nanoporous aluminum showed high variations. Overall, nanoporous aluminum showed promising results as it can be used to coat conveyor belts. However, compatibility with cleaning detergent and sensitivity to scratches must be further investigated. Overall, it can be concluded that cleanability is not only influenced by surface roughness, but also by the overall surface finish, scratches, and defects.

**Supplementary Information:**

The online version contains supplementary material available at 10.1007/s13197-023-05778-0.

## Introduction

In Western societies, all kinds of food exist abundantly and in a great range of variety. Due to globalization, long-distance commerce in raw and prepared foods is becoming very common. Thus, more emphasis is now put on the safety, quality, and long shelf life of these foods, especially meat and meat products (Kreyenschmidt and Ibald 2012). In 2015, the WHO published the first-ever report showing that each year, 1 out of 10 people get ill from food contaminated with microbial or chemical agents, resulting in 600 million illnesses, 420 000 deaths, and the loss of 33 million healthy years of life in the world (WHO 2015). A significant number of foodborne diseases can be associated with animal products (meat, dairy), which can be explained by intensive livestock farming, mass production, and extended distribution channels (Jones et al. 2013). Temperature conditions along the chain and hygienic conditions during processing are crucial to reduce cross-contamination and microbial growth and thus to increase food safety (Albrecht et al. 2020; Barbut 2015). Cross-contamination is responsible for many foodborne diseases outbreaks caused by pathogenic bacteria that can occur due to contaminated equipment and inadequate use of surface materials in food processing facilities due to insufficient design or hygiene measures (Griffith et al. 2015; Teixeira et al. 2007). Bacterial adhesion to surfaces is influenced by multiple intrinsic and extrinsic factors, such as characteristics of different bacterial species, surface properties, contact time, and the presence of other bacteria or biofilms (Gkana et al. 2017; Hüwe 2018). Frequent and adequate cleaning and disinfection processes, adapted to specific food matrices, are of high importance to prevent bacterial colonization on surfaces (Gram et al. 2007). However, well-established hygienic operations performed in the food industry are often not completely effective. Møretrø et al. (2013) showed only a low bacterial reduction on food contact surfaces after the application of several disinfectants.

Besides cleaning and disinfection, different approaches have been developed in recent years to reduce the risk of cross-contamination, such as antimicrobial surfaces (Braun et al. 2017) and hygienic design principles of machinery (Hofmann et al. 2018). The idea of hygienic equipment design emerged initially in the dairy industry by developing the specific 3A Sanitary Standards, which the meat industry has started following in recent years (Barbut 2015; Fortin 2011). Hygienic design and selection of construction materials (e.g., conveyor belts, trays, cutting surfaces) are essential to enhance the safety of food as well as minimize bacterial attachment and biofilm formation (Barbut 2015). Generally, a hygienic surface should be inert and easy to clean; thus, stainless steel is mainly used in the construction of equipment in the food industry (Hofmann et al. 2018). Besides that, polymeric materials are also used where needed. However, some can be vulnerable to chemical abrasion and temperature fluctuation (Hofmann et al. 2018). The average roughness value R_a_ is generally associated with cleanability and the hygienic status of food contact surfaces. The European Hygienic Engineering and Design Group and the American Meat Institute (AMI) recommend a R_a_ value of a maximum of 0.8 µm (Hofmann et al. 2018; Schmidt et al. 2012).

Besides conventionally used stainless steel and polymer surfaces, several new materials have been recently developed in order to reduce potential microorganism attachment and biofilm formation, to increase the ease of cleanability, and hence minimize cross-contamination during processing. Especially nanostructured surfaces are showing promising properties to control bacterial adhesion and biofilm formation. In that case, material characteristics are modified by nanoscaling, which can also improve mechanical strength and thermal resistance (Chausali et al. 2022; Souza et al. 2021). Furthermore, nanoscaled surfaces have the potential of being antimicrobial based on their material of construction-related physicochemical characteristics (Khezerlou et al. 2018).

A wide range of conveyor belt materials and surface topographies have already been developed. But so far, there are few reports providing comprehensive comparisons concerning the cleanability of innovative surfaces from different materials and surface structures. Information in this field is of great importance, as conveyor belts (with their large surface areas) can be hotspots for cross-contamination.

Thus, the objectives of this study were to investigate and compare the cleanability of different conveyor belt materials consisting of thermoplastic polymers, stainless steels, and new aluminized surfaces with unique nanostructures. The cleanability was tested by contamination with a known bacterial culture, followed by cleaning with either water or an alkaline solution. In addition, cleanability properties were assessed by scanning electron microscopy (SEM) examination.

## Materials and methods

### Experimental design

Different conveyor belts currently used by the meat and food industry, or surfaces with the potential to be implemented in the future, were investigated regarding their cleanability and microstructure. A total of 20 different thermoplastic polymers, stainless steels (with different surface finishes), and aluminized nanoporous surfaces (with highly uniform size features and distribution profiles) were compared. Two different experimental cleaning setups were used: (1) cleaning with distilled water and (2) with alkaline foam detergent. A suspension of *Pseudomonas fluorescens* was used to soil the surfaces*.* Reference surfaces (samples of each material) were also exposed to *Ps. fluorescens* but remained uncleaned. The results are reported as the reduction of microbial counts by each cleaning process compared to the reference surfaces. Based on the results, a ranking of cleanability was established. Microscopy was also performed to characterize the surfaces.

### Sample surfaces

A total of 20 sample surfaces, consisting of 11 thermoplastic polymers, 4 stainless steel, and 5 aluminized nanostructured surfaces, were investigated (Table 1). The nanoporous materials are made of an aluminum base material with a top upper layer that was transformed into alumina by applying a high electrostatic charge. Samples were supplied by Marel Poultry B.V., (Boxmeer, Netherlands) to the University of Bonn for testing.

**Table 1:** Sample surfaces of thermoplastic polymers, stainless steels, and aluminized nanoporous materials tested for cleanability

Surface ID	Material	Surface characteristics
Polymers		Surface structures
A	Polyurethane	Flexible, smooth, coated web
B	Polyurethane	Fibrous, woven, 3 mm patterns
C	Polyurethane	Flexible, trapezoid immersions
D	Polyurethane	Hard, rough, ridged, hinged
E	Polyurethane	Flexible, smooth
F	Polyoxymethylene	Stiff, rough
G	Polyoxymethylene	Smooth, ridged
H	Polyoxymethylene	Ridged, hinged
I	Polyoxymethylene	Ridged, hinged
J	Polyoxymethylene	Hard, smooth, hinged
K	Polyolefins	Flexible, woven

Stainless steel		Finish	R_a_ [µm]
SS1	Type 1.4162 duplex 2204 (> 20% Cr, 5% Ni, 3% Mo)	Drum-polished	0.300–0.500
SS2	Type 1.4162 duplex 2204 (> 20% Cr, 5% Ni, 3% Mo)	Glass-bead blasted; rough	1.8–2.1
SS3	Type 1.4162 duplex 2204 (> 20% Cr, 5% Ni, 3% Mo)	Electro-polished	0.595
SS4	Type 1.4162 duplex 2204 (> 20% Cr, 5% Ni, 3% Mo)	Glass-bead blasted; fine	0.8

Nanoporous aluminum		Interpore distance [nm]	Pore diameter [nm]	Length[µm]
#25	Al_2_O_3_	65	25	10
#40	Al_2_O_3_	125	40	10
#90	Al_2_O_3_	125	90	10
#150	Al_2_O_3_	365	150	10
#300	Al_2_O_3_	365	300	10

The thermoplastic and stainless steel samples were cut into 25 × 25 mm (6.25 cm^2^) coupons. The aluminized surfaces were provided as discs with a diameter of 50 mm. The discs were cut into four equal pieces with a surface area of 4.91 cm^2^ each. Before each trial, all samples were sterilized by boiling in distilled water for 10 min and disinfected with a 70% ethanol swab (Lohmann Laborservice GmbH, Marxen, Germany).

### Cleanability tests

**Fig. 1:**
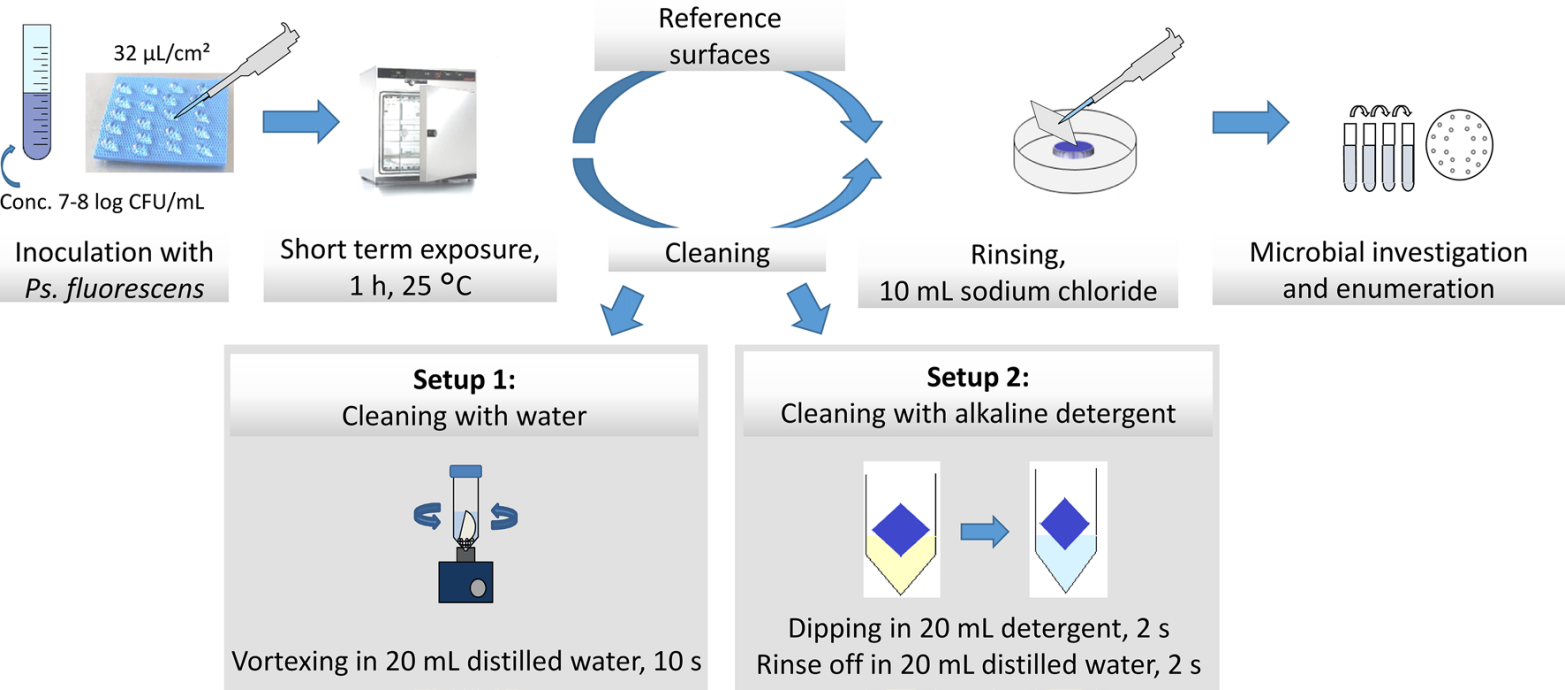
Schematic overview of the experimental setup of the cleaning tests with water and an alkaline detergent

Figure 1 provides a schemed overview of the steps applied.

#### Inoculation

Bacterial cultures for the inoculation of the surfaces were prepared (Fig. 1). *Ps. fluorescens* (DSM 50091) was selected as the test organism, as it is a typical spoilage organism for fresh meat, also known to have a strong adhesion to surfaces (Wang et al. 2018). For preparing the inoculation, cryo-conserved bacteria stock was transferred in 10 mL of nutrient broth (Merck KGaA, Darmstadt, Germany) and incubated for 24 h at 25 °C. Prior to the trial, 1 mL of the cultivated suspension was transferred in 9 mL of a 0.85% sodium chloride (Oxoid GmbH, Wesel, Germany) and 0.1% tryptone (VWR International GmbH, Darmstadt, Germany) solution to obtain a controlled bacteria culture of approximately log_10_ 8 CFU/mL.

For both cleaning setups, each surface material was investigated in 9–15 repetitions, depending on the specific surface material. The sterilized samples were placed in sterile Petri dishes, and each was inoculated with 0.2 mL bacteria suspension by dispersing 20 drops of 0.01 mL with a multi pipette (Eppendorf AG, Hamburg, Germany) to obtain an inoculation of 32 µL/cm^2^. The drops were spread at equal distances on the surface to achieve a consistent spread. The aluminized surfaces were inoculated with 0.16 mL bacteria suspension by dispersing 16 drops of 0.01 mL, i.e., adapted to the smaller surface. Consequently, an inoculation of 32 μL^/^cm^2^ was obtained on each surface. The reference surfaces of each material were inoculated similarly. The sample and reference surfaces were stored for 1 h at 25 °C in an incubator (Memmert GmbH + Co. KG, Schwabach, Germany).

#### Cleaning

After 1 h, cleaning was performed with (1) distilled water or (2) 0.1% alkaline detergent solution (Orbin VR-S. Büfer Reinigungssysteme GmbH & Co. KG, Oldenburg, Germany), which is a commercial alkaline foam without active chlorine, and with pronounced detaching power to remove heavy grease and protein soiling (ingredients: sodium hydroxide, 2-phosphonobutane-1, 2, 4-tricarboxylic acid, amines, coco alkyldimethyl, N-oxides, phosphonates, anionic surfactants, non-ionic surfactants). The common use level for routine food processing equipment cleaning is 1–5% with an exposure time of 10–20 min. The concentration of detergent and exposure time to the surfaces were adjusted to a laboratory scale and also to allow differentiation among the surfaces.

When cleaning with water, the incubated samples were transferred into tubes with 20 mL of distilled water and cleaned by vortexing for 10 s and then returning to the Petri dishes. When using the detergent, the incubated samples were dipped in tubes with 20 mL of alkaline detergent (completely covered) for 2 s. The samples were then transferred into tubes with 20 mL of distilled water for 2 s and then returned to the Petri dishes. The inoculated reference samples remained uncleaned in both procedures and subjected to microbial analysis without the cleaning step, i.e., used for calculating the microbial reduction of the cleaned samples.

#### Microbial analysis

The cleaned samples and reference samples were later rinsed ten times manually by a single channel pipette with 1 mL of a 0.85% sodium chloride (Oxoid GmbH, Wesel, Germany) and 0.1% tryptone (VWR International GmbH, Darmstadt, Germany) solution (total 10 mL) in order to detach the bacteria.

For microbial analysis, 1 mL of the rinsing solution was transferred into 9 mL of sodium chloride and tryptone solution, and a tenfold dilution series was prepared. Appropriate dilutions were transferred on to growth medium (Merck KGaA plate count agar, Darmstadt, Germany) in Petri dishes. The bacterial count was determined by the spread-plate technique, incubated at 25 °C for 48 h. Each sample was plated and enumerated in duplicate. Data were reported as log_10_ CFU/cm^2^ due to the varying sizes of the samples.

### Microscopy

A scanning electron microscope (FEI Quanta FEG 250—Thermo Fisher Scientific, Hillsboro, ONT, Canada) was used to examine the microstructure of the surfaces. The plastic samples were first coated with gold–palladium (to allow electron conductivity/reflection), while the metal samples did not require coating. Samples were tilted at 25° to enhance the view of the surfaces’ topography. The samples were viewed (under 10 kV) and photographed at different magnifications (i.e., a higher magnification was used for the aluminum nanostructures).

### Data analysis and statistics

The reduction of bacterial loads was calculated by subtracting the logarithmic value of the calculated viable counts on the cleaned samples from the viable counts on the reference samples for each sample, as shown in the following equation (Bacterial counts are reported as log_10_ CFU/cm^2^).

$${\text{Bacterial}}\;{\text{reduction}}\left[ {\log_{10} \,{\text{CFU/cm}}^{{2}} } \right] = \log_{10} \,{\text{CFU/cm}}^{{2}} \left( {\eta_{{\text{Re}}} } \right) - \log_{10} \,{\text{CFU/cm}}^{{2}} \left( {\eta_{Sa} } \right)$$

where *ƞ*_Re_ = bacterial concentration on reference material and *ƞ*_Sa_ = bacterial concentration on sample material

The cleanability of the surfaces is expressed as the percentage of reduction of the bacterial concentration. Median values, as well as first and third quartiles of percentage reduction in bacterial counts are presented as box plots. Differences in the cleanability of the surfaces were analyzed for significance using the Kruskal–Wallis test by rank for independent samples with pairwise comparisons. The significance level was defined as *p* ≤ 0.05 and *p* ≤ 0.01 for a highly significant difference. Data analysis was conducted with SPSS Statistics 25 (IBM Corp. 1989, 2017, New York, USA).

## Results

### Cleanability of surfaces cleaned with water (Setup 1)

**Fig. 2:**
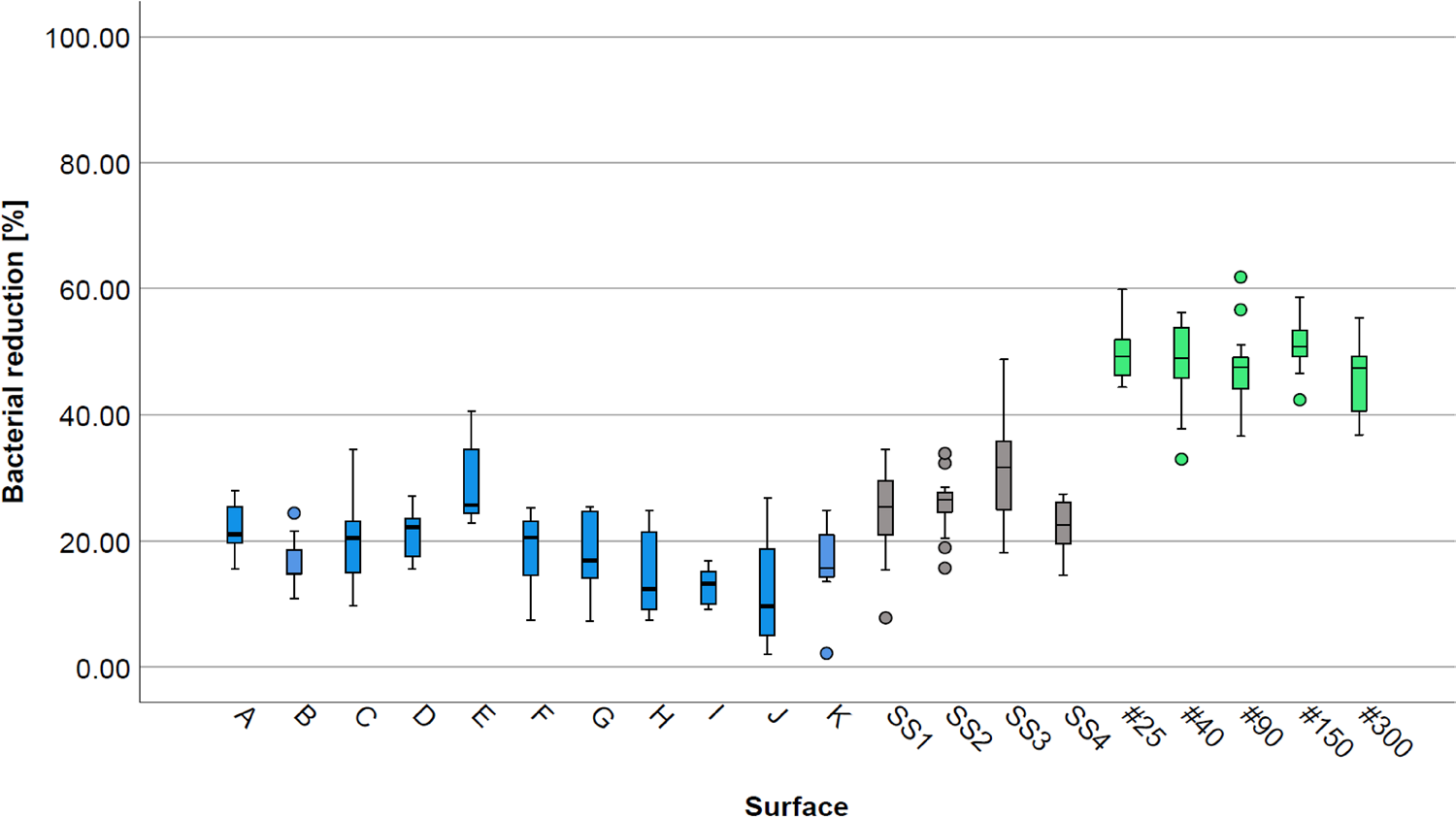
Box plots of bacterial reduction [%] on thermoplastic (A, B, D, E, K: n = 9; C, F, G, H, I, J: n = 12), stainless steel (n = 15) and aluminized nanoporous surfaces (n = 15), inoculated with Ps. fluorescens for 1 h at 25 °C, after cleaning with water

The bacterial reduction rates of *Ps. fluorescens* after surface cleaning, expressed as percentages are shown in Fig. 2. Considering all investigated surfaces, the bacterial reduction rates of *Ps. fluorescens* showed differences in medians of up to 40% (9.70–50.78%). Nanoporous aluminum surfaces showed the overall highest bacterial reduction rates, followed by the group of stainless steels. Thermoplastic surfaces showed the lowest reduction rates, ranging from 9.70% to 25.69%, with the lowest bacterial reduction for polyoxymethylene surface J and the highest for polyurethane surface E. Comparing the polyurethane and polyoxymethylene groups, higher median reduction rates for polyurethane were observed for cleaning with water. The reduction of the bacterial count on stainless steel samples ranged from 22.49% for glass-bead blasted (fine) (SS4) to 30.63% for electro-polished (SS3) stainless steel. Aluminized nano surfaces showed the overall highest reduction rates for cleaning with water ranging from 47.32% to 50.78% for the aluminum with pore diameters of 300 nm (#300) and 150 nm (#150), respectively. The results of the Kruskal–Wallis test by rank, used for values significance analysis, have been adjusted by the Bonferroni correction for multiple tests. The respective probability value (*p*-value) indicates the significance level. A significant difference is reported with a *p*-value < 0.05; a highly significant difference with a *p*-value of < 0.01. The variation in reduction rates showed no clear trend among the three different material groups.

All aluminized nanoporous surfaces showed significantly higher bacterial reduction rates with a *p*-value of < 0.01 than the following thermoplastic surfaces: Polyurethanes B and C, all polyoxymethylene surfaces (F, G, H, I, J) and polyolefins surface K. Significant higher bacterial reduction rates were shown for nanostructured surfaces #90 and #300, when compared to glass-bead blasted fine stainless steel SS4. Furthermore, surfaces #25, #40, and #150 showed highly significantly higher reduction rates when compared to SS4. The difference in reduction rates of nanoporous aluminum #150 was highly significant when compared to surfaces A and D and SS1; and significant when compared to SS2. Nanoporous surfaces #25 and #40 showed significantly higher reduction rates when compared to thermoplastics A, D, and stainless steel SS1. Electropolished stainless steel (SS3) showed significantly higher bacterial reduction than polyoxymethylene surface J. There was no significant difference in *Ps. fluorescens* reduction within the groups of nanocoated surfaces, stainless steels, and polymer surfaces. Detailed results for *p*-values of the statistical analysis with the Kruskal–Wallis test can be found in the supplementary files.

### Cleanability of surfaces cleaned with alkaline detergent (Setup 2)

**Fig. 3:**
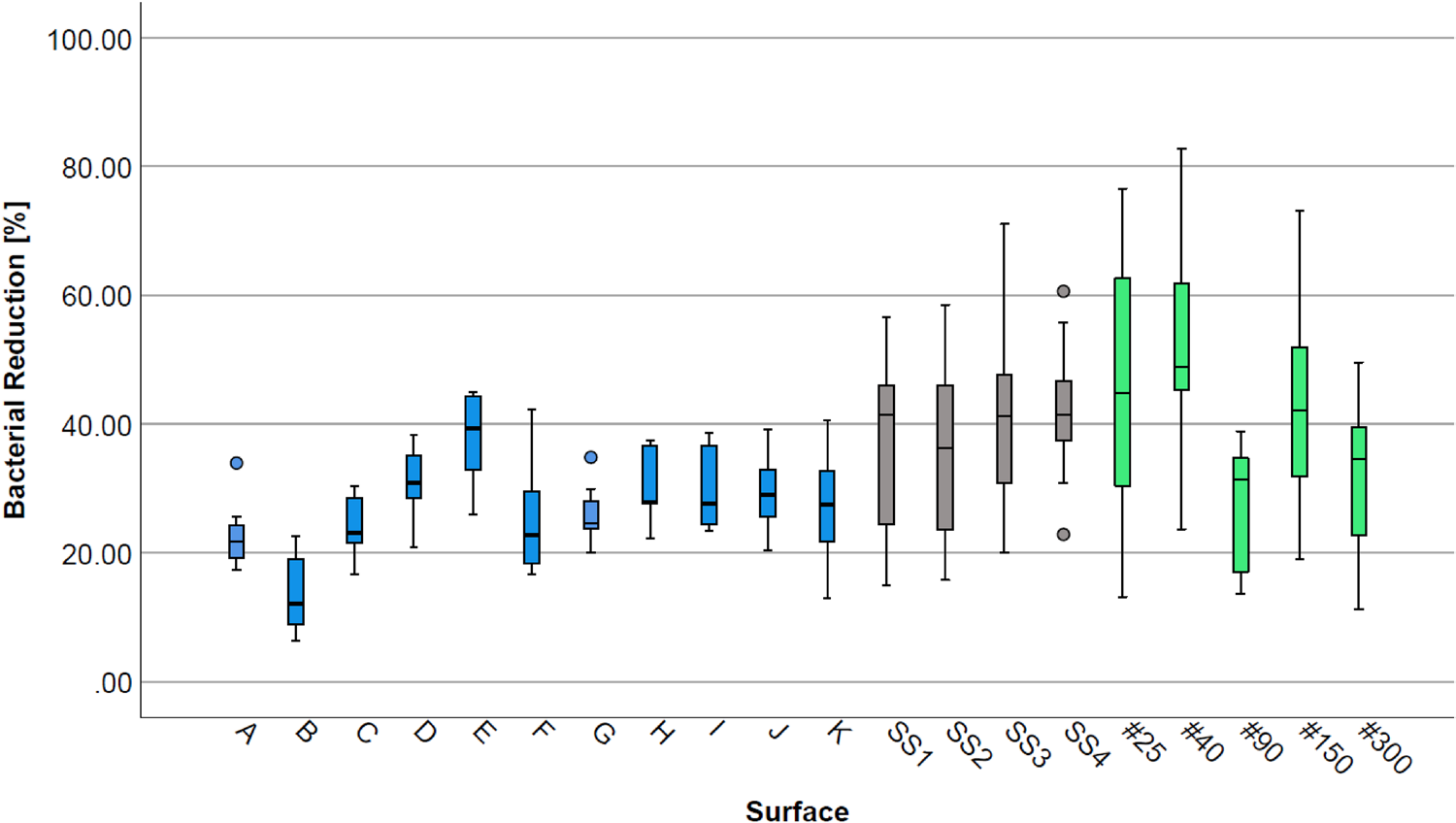
Box plots of bacterial reduction [%] on thermoplastic (A, B, D, E, K: n = 9; C, F, G, H, I, J: n = 12), stainless steel (n = 15) and aluminized nanoporous surfaces (n = 15), inoculated with *Ps. fluorescens* for 1 h at 25 °C, after cleaning with 0.1% alkaline detergent

Figure 3 shows the percentual reduction rates, on surfaces, after cleaning with an 0.1% alkaline detergent solution. The medians of bacterial reduction rates for all surfaces were between 12.18% and 48.79% (polyurethane surface B and nanoporous aluminum #40, respectively). The variation in the individual reduction rates was highest for the group of nanoporous aluminum, followed by stainless steel, and lowest for polymer surfaces. The results in bacterial reduction in this setup also indicated that nanoporous aluminum surfaces showed the highest median reduction rates, with 31.48% for aluminum with a pore diameter of 90 nm (#90) to 48.79% (#40). Thermoplastic polymers showed the lowest individual median reduction rates, with a maximum reduction rate of 39.38% for polyurethane surface E. The stainless steel surfaces overall showed median reduction rates between 36.34% for glass-bead blasted (rough) (SS2) and 41.33% for drum-polished stainless steel (SS1). The reduction rates achieved by cleaning the nanoporous aluminum ranged from medians of 17.46% to 48.79%, with the lowest reduction seen in surface #90 and the highest in #40.

The results of statistical analyses with significant values calculated by the Kruskal–Wallis test and the Bonferroni correction showed that bacterial reduction on polyurethane surface B was highly significantly lower than on electropolished stainless steel (SS3) and glass-bead blasted fine (SS4) surfaces, as well as on nanoporous aluminum samples #25, #40 and #150. Furthermore, polyurethane surface B showed a significantly lower bacterial reduction than polyurethane E and drum-polished stainless steel (SS1). Nanoporous aluminum with a pore diameter of 40 nm (#40) showed highly significant higher bacterial reduction than polyurethanes A, C, and polyoxymethylene F as well as significantly higher reduction than polyoxymethylenes G and K. Except for a significantly (*p* < 0.05) higher reduction rate seen in surface #40 compared to #90, no significant differences were revealed amongst the nanoporous aluminum surfaces. Additionally, no significant differences were found between stainless steels and aluminum surfaces as well as within the group of stainless steel surfaces. Detailed results for *p*-values of the statistical analysis with Kruskal–Wallis test can be found in the supplementary files.

### Investigation of surface topography by scanning electron microscopy

**Fig. 4:**
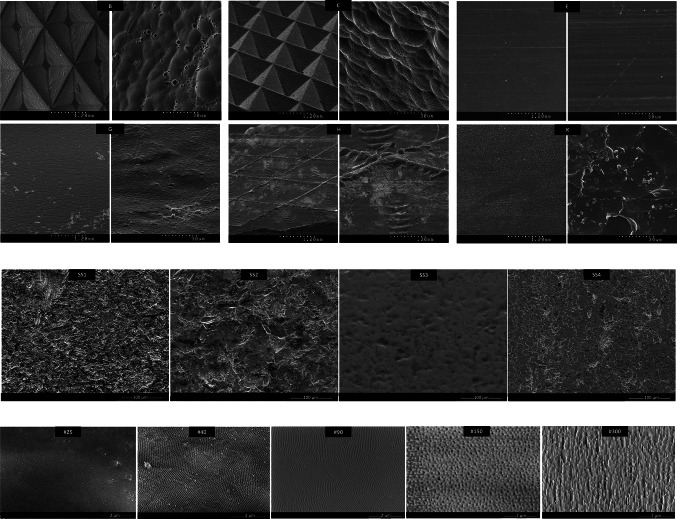
Comparison of selected surfaces using a scanning electron microscope (top set-plastic belts; middle-stainless steel; bottom-nanostructured aluminum surfaces). Magnification bars are indicated within each micrograph. * Each boxplot reflects the 25th, 50th (Median), and 75th percentile. Box whiskers represent the minimum and maximum of the distribution. Dots reflect mild outliers

Topographies of the samples can be seen both with the naked eye (mm range) and at high resolution by scanning electron microscopy (µm range). Photographs of clean samples were taken and shown in Fig. 4. Varying magnifications were used according to the different surfaces topographies.

SEM revealed highly visible differences in the surface compositions of polymer surfaces. Polyurethane surface E showed smooth properties of the high-resolution surface, while surfaces B and C (also polyurethanes) showed strong immersions and distinct irregularities. Surfaces G, H, and K, selected as samples whose cleanability results are in a medium range, also showed slight to increased surface roughness, especially considering a magnification of 30 µm.

Visible differences in characteristics of glass bead blasted rough (SS2) and fine (SS4) surfaces were revealed on a magnification level of 100 µm. Electropolished surface SS3 showed partially smoother areas when compared to SS1, SS2, and SS4.

Photographs taken of nanoporous aluminum with a magnification of 2 µm indicated the differences in interpore distances, increasing from surfaces #25 to #300 with distances from 65 to 365 nm (see also Table 1).

## Discussion

This study provides an overall analysis of a wide range of plastic and metal materials used as conveyor belts/contact surfaces in the meat/food industry; these include thermoplastic polymers, stainless steels, and prototypes of nanoporous aluminum surfaces with the possibility of industrial implementation. Surface cleanability under laboratory conditions with water showed higher cleanability rates for nanoporous aluminum compared to stainless steel and polymer surfaces. Cleaning with 0.1% alkaline detergent also showed high reduction rates for nanoporous aluminum but with high variations. In general, the variance of the reduction rates was higher when compared to water cleaning for most of the investigated surfaces. Thermoplastic polymers showed the overall lowest cleanability rates in both cleaning setups regarding all investigated surfaces and their surface materials. However, cleanability rates of the individual thermoplastic surfaces were higher when they were cleaned with alkaline detergent, except for the woven, shaped polyurethane surface B. This surface showed a low bacterial reduction in both setups. Micrographs of the materials revealed surface irregularities on a microscale level. In this specific case, these fibrous structures can facilitate bacterial attachment and long-term adhesion; thus, surface B does not seem suitable for contact with raw meat. Furthermore, the investigations revealed no distinct difference among the polymer materials (polyurethane, polyoxymethylene, and polyolefins). Flexible, smooth polyurethane E can be identified as the polymer with the highest rate of bacteria reduction in both cleaning setups. By cleaning with water, surface E is the only polymer surface that showed no significant difference in cleanability compared to nanoporous aluminum. However, compared to stainless steel and nano surfaces, polyurethane E showed overall lower cleanability tendencies under both setups. In general, plastics possess various advantages for use in conveyor belts, such as flexibility, hydrophobicity, and corrosion resistance; however, porosity can enable microorganisms to attach and persist (Kold and Silverman 2016). The overall macro patterns on the plastic surfaces (e.g., ridges, hinges, woven structures used to prevent slippage, connect pieces, and enhance strength) which are clearly visible on SEM micrographs, can provide a suitable environment for bacteria to attach. It is known that different materials, e.g., stainless steel and polymers, with various surface characteristics, can influence the rate of bacterial adhesion and biofilm formation (Srey et al. 2013); however, there are no consistent results concerning exact material preferences, as attachment processes are very complex.

Concerning stainless steel, all surfaces showed good cleanability when cleaned with an alkaline detergent. For both cleaning setups, no significant differences between the four stainless steel finishes could be detected, even though the R_a_ values were different. An irregular surface topography in the glass-bead blasted rough and fine surfaces (SS2, SS4) can be observed (Fig. 4). Schmidt et al. (2012) explained that glass bead blasting of surfaces is less applied for food contact surfaces due to the rather irregular shape after treatment. Electropolished stainless is known as the most sanitary stainless steel, as it has low roughness values (Frank and Chmielewski 2001), and in this study, the R_a_ value was 0.595 µm. Drum-polished stainless steel (SS1) revealed the lowest R_a_ (0.3–0.5 µm); however, it showed cleanability values comparable to the other stainless steels. Flint et al. (2000) found a maximum bacterial adhesion at a R_a_ value of 0.9 µm. However, a roughness higher than 0.8 μm is acceptable by compensating it with suitable adjustments like the frequency of cleaning actions (Hofmann et al. 2018). The study results concerning stainless steel surfaces show that a high cleanability cannot be directly associated with individual R_a_ values. Boyd et al. (2001) and Milledge (2010) also mentioned that the average roughness value is not the only decisive criterion for the cleanability of a surface, as it does not consider the overall topography and cannot be set in direct relation to the finish. The maximum roughness (Rmax) and the averaged roughness depth (RZ/DIN) include the maximum and mean peak-to-valley height within surfaces and thus can be more appropriate indicators for the cleanability performance of surfaces (Frank and Chmielewski 2001). In addition, these maximum roughness limits can differentiate desired roughness from unintentional surface scratches, which can also affect the cleanability rate of used equipment (Bobe and Wildbrett 2006). Defects and scratches, which occur inevitably during the lifetime of food contact surfaces, can change the average roughness as well as affect the cleanability and the hygienic status (Frank and Chmielewski 2001).

The best rates of cleanability were achieved for the nanoporous aluminum, especially in setup 1. It indicates that nanostructures can be a promising new candidate for conveyor belt surfaces. Furthermore, both cleaning setups showed that surfaces with pore diameters of 90 and 300 nm had lower cleanability rates; however, no significant difference was revealed when compared to the other nanoporous surfaces. The bacterial attachment properties of nanoscaled surfaces can vary depending on the specific pore diameters. It was shown that nano surfaces with small pore diameters of 15 and 25 nm had a significantly lower bacterial attachment and biofilm formation of *Listeria innocua* and *Escherichia coli* (Feng et al. 2014). However, it is crucial to differentiate between the properties of bacterial attachment and cleanability. In any case, it can be assumed that pore size, as well as other physical and chemical properties of nanoscaled surfaces influence bacterial adhesion and cleaning.

The lower cleanability rates seen in setup 2 when compared to setup 1, as well as the high variations within individual surfaces, can be explained by a variety of factors. One may be that aluminum is highly sensitive to chemical agents. Treatment with an alkaline cleaner can alter the microstructure of aluminum surfaces and alloys and by that change to more hydrophilic surface characteristics (Chen et al. 2021; Tiringer et al. 2017). Furthermore, irregularities in the material can possibly lead to high variations in the results. In addition, the cleaning method (e.g., dipping the sample into an alkaline detergent solution for 2 s) may be sensitive to errors, which could cause a high standard deviation. It should be also mentioned that higher variations in cleaning with alkaline detergents were also seen for stainless steel (41.57–58.33%) when compared to cleaning results with water (12.95–30.55%). Overall, additional studies employing different cleaning agents and methods will be required to get more information about the effect on cleaning efficiencies. However, the hydrophobic surface character, the minor bacterial attachment, and the microbial inactivation potential of nanostructured surfaces can lead to an enhanced hygienic status of the surface and a higher cleanability compared to stainless steel and thermoplastic surfaces (Khezerlou et al. 2018; Peters et al. 2016). The antimicrobial effect of nanoscaled aluminum has been shown in previous studies (Feng et al. 2014; Mukherjee et al. 2011). However, additional challenges exist concerning the practical application of aluminum surfaces in the food industry. Overall, aluminum is a soft metal sensitive to scratches, which can alter nanostructures and thus influence bacterial attachment and cleanability. The scratch resistance is thus of special importance for food contact surfaces, as it prevents a lack of cleanability and leads to a longer life cycle. In this context, durability and sustainability are important factors concerning the long-term usage of equipment. Furthermore, welding processes are challenging due to the fragile nature of aluminum (Feng et al. 2014). Nevertheless, nanoscaled surfaces provide promising applications for food contact surfaces due to their ease of cleaning. In any case, the potential of toxicity and migration of nanoscaled materials to food must be considered prior to application.

## Conclusion

In the study, it was shown that cleanability strongly depends on the material used. The application of conveyor belt surfaces with high cleanability can improve overall hygiene management in the meat/food industry by preventing cross-contamination and reducing health hazards to the consumer. Regarding the cleaning media, water and detergent can be reduced, which leads to the protection of the material due to less attrition and overall, to a more sustainable, cost and time-effective process.

Further research should focus on the relations between cleanability and roughness parameters (Rmax and RZ(DIN)) in order to consider the effects of surface topography/potential scratches and defects on cleanability. Furthermore, surface cleanability should also be investigated with sight on long-term material stability as well as the potential formation of biofilms.

## Supplementary Information

Below is the link to the electronic supplementary material.

Supplementary file1 (DOCX 30 KB)

## Data Availability

All data generated or analysed during this study are included in this published article and its supplementary files.

## References

[CR1] Albrecht A, Ibald R, Raab V, Reichstein W, Haarer D, Kreyenschmidt J (2020). Implementation of time temperature indicators to improve temperature monitoring and support dynamic shelf life in meat supply chains. J Packag Technol Res.

[CR2] Barbut S (2015) The science of poultry and meat processing. University of Guelph, Guelph, Ontario, Canada. Free download at: https://www.poultryandmeatprocessing.com/

[CR3] Bobe U, Wildbrett G (2006). Anforderungen an Werkstoffe und Werkstoffoberflächen bezüglich Reinigbarkeit und Beständigkeit. Chem Ing Tec.

[CR4] Boyd RD, Cole D, Rowe D, Verran J, Paul AJ, West RH (2001). Cleanability of soiled stainless steel as studied by atomic force microscopy and time of flight secondary ion mass spectrometry. J Food Prot.

[CR5] Braun C, Dohlen S, Ilg Y, Brodkorb F, Fischer B, Heindirk P, Kalbfleisch K, Richter T, Robers O, Kreyenschmidt M, Lorenz R, Kreyenschmidt J (2017). Antimicrobial activity of intrinsic antimicrobial polymers based on poly((tertbutyl-amino)-methyl-styrene) against selected pathogenic and spoilage microorganisms relevant in meat processing facilities. J Antimicrob Agents.

[CR6] Chausali N, Saxena J, Prasad R (2022). Recent trends in nanotechnology applications of bio-based packaging. J Agric Food Res.

[CR7] Chen S-Y, Huang C-Y, Lin C-S (2021). Microstructure inhomogeneity of the constituent particles of 7075–T6 aluminum alloy after alkaline cleaning and desmutting. Corros Sci.

[CR8] Feng G, Cheng Y, Wang S-Y, Hsu LC, Feliz Y, Borca-Tasciuc DA, Worobo RW, Moraru CI (2014). Alumina surfaces with nanoscale topography reduce attachment and biofilm formation by Escherichia coli and Listeria spp. Biofouling.

[CR9] Flint SH, Brooks JD, Bremer PJ (2000). Properties of the stainless steel substrate, influencing the adhesion of thermo-resistant streptococci. J Food Eng.

[CR10] Fortin ND (2011) Regulations on the hygienic design of food processing factories in the United States This chapter is derived from Food Regulation: Law, Science, Policy, and Practice, John Wiley & Sons, Inc. (2009), by Neal Fortin and is used with the permission of the publisher. In: Hygienic Design of Food Factories. Elsevier, Netherlands pp 55–74

[CR11] Frank JF, Chmielewski R (2001). Influence of surface finish on the cleanability of stainless steel. J Food Prot.

[CR12] Gkana E, Chorianopoulos N, Grounta A, Koutsoumanis K, Nychas G-JE (2017). Effect of inoculum size, bacterial species, type of surfaces and contact time to the transfer of foodborne pathogens from inoculated to non-inoculated beef fillets via food processing surfaces. Food Microbiol.

[CR13] Gram L, Bagge-Ravn D, Ng YY, Gymoese P, Vogel BF (2007). Influence of food soiling matrix on cleaning and disinfection efficiency on surface attached Listeria monocytogenes. Food Control.

[CR14] Griffith A, Neethirajan S, Warriner K (2015). Development and evaluation of silver zeolite antifouling coatings on stainless steel for food contact surfaces. J Food Saf.

[CR15] Hofmann J, Åkesson S, Curiel G, Wouters P, Timperley A (2018) Hygienic design principles. EHEDG Guidelines

[CR16] Hüwe C (2018). Intrinsically antimicrobial active polymers to improve the hygienic conditions during processing and preparation of fresh meat.

[CR17] Jones BA, Grace D, Kock R, Alonso S, Rushton J, Said MY, McKeever D, Mutua F, Young J, McDermott J, Pfeiffer DU (2013). Zoonosis emergence linked to agricultural intensification and environmental change. Proc Natl Acad Sci USA.

[CR18] Khezerlou A, Alizadeh-Sani M, Azizi-Lalabadi M, Ehsani A (2018). Nanoparticles and their antimicrobial properties against pathogens including bacteria, fungi, parasites and viruses. Microb Pathog.

[CR19] Kold J, Silverman C (2016). Conveyors used in the food industry. Handbook of hygiene control in the food industry.

[CR20] Kreyenschmidt J, Ibald R, Nicoli M (2012). Modeling shelf life using microbial indicators. Shelf Life Assessment of Food.

[CR21] Milledge J (2010). The cleanability of stainless steel used as a food contact surface: an updated short review. Food Sci Technol.

[CR22] Møretrø T, Langsrud S, Heir E (2013). Bacteria on meat abattoir process surfaces after sanitation: characterisation of survival properties of listeria monocytogenes and the commensal bacterial flora. Adv Microbiol.

[CR23] Mukherjee A, Sadiq IM, T.C. P, Chandrasekaran N,, Méndez-Vilas A (2011). Antimicrobial activity of aluminium oxide nanoparticles for potential clinical applications. Science against microbial pathogens: communicating current research and technological advances.

[CR24] Peters RJ, Bouwmeester H, Gottardo S, Amenta V, Arena M, Brandhoff P, Marvin HJ, Mech A, Moniz FB, Pesudo LQ, Rauscher H, Schoonjans R, Undas AK, Vettori MV, Weigel S, Aschberger K (2016). Nanomaterials for products and application in agriculture, feed and food. Trends Food Sci Technol.

[CR25] Schmidt RH, Erickson DJ, Sims S, Wolff P (2012). Characteristics of food contact surface materials: stainless steel. Food Prot Trends.

[CR26] Souza AG, Ferreira RR, Paula LC, Mitra SK, Rosa DS (2021). Starch-based films enriched with nanocellulose-stabilized Pickering emulsions containing different essential oils for possible applications in food packaging. Food Packag Shelf Life.

[CR27] Srey S, Jahid IK, Ha S-D (2013). Biofilm formation in food industries: a food safety concern. Food Control.

[CR28] Teixeira P, Silva SC, Araújo F, Azeredo J, Oliveira R, Méndez-Vilas A (2007). Bacterial adhesion to food contacting surfaces. Communicating current research and educational topics and trends in applied microbiology.

[CR29] Tiringer U, Kovač J, Milošev I (2017). Effects of mechanical and chemical pre-treatments on the morphology and composition of surfaces of aluminium alloys 7075–T6 and 2024–T3. Corros Sci.

[CR30] Wang H, Cai L, Li Y, Xu X, Zhou G (2018). Biofilm formation by meat-borne Pseudomonas fluorescens on stainless steel and its resistance to disinfectants. Food Control.

[CR31] WHO (2015). WHO estimates of the global burden of foodborne diseases.

