# Supplementary material for: Microbial investigation of cleanability of different plastic and metal surfaces used by the food industry
Source: J Food Sci Technol. 2023 Jul 6;60(10):2581–90. doi: 10.1007/s13197-023-05778-0 (PMC10439085; doi:10.1007/s13197-023-05778-0)
Supplement: Supplementary file 1 — Supplementary file1 (DOCX 30 KB) [file 13197_2023_5778_MOESM1_ESM.docx]

## Supplementary Material

**Fig. ESM1** Statistical analysis of bacterial reductions on investigated surfaces after cleaning with water, application of Kruskal-Wallis test adjusted by Bonferroni correction, for p = 0.05 and for p = 0.01

**Fig. ESM2** Statistical analysis of bacterial reductions on investigated surfaces after cleaning with 0.1% alkaline detergent, application of Kruskal-Wallis test adjusted by Bonferroni correction, for *p* = 0.05 and for *p =* 0.01
